# A Prospective Study on the Fermentation Landscape of Gaseous Substrates to Biorenewables Using *Methanosarcina acetivorans* Metabolic Model

**DOI:** 10.3389/fmicb.2018.01855

**Published:** 2018-08-24

**Authors:** Hadi Nazem-Bokaee, Costas D. Maranas

**Affiliations:** Department of Chemical Engineering, The Pennsylvania State University, University Park, PA, United States

**Keywords:** gas fermentation, metabolic modeling, CH_4_, CO, CO_2_, *M. acetivorans*

## Abstract

The abundance of methane in shale gas and of other gases such as carbon monoxide, hydrogen, and carbon dioxide as chemical process byproducts has motivated the use of gas fermentation for bioproduction. Recent advances in metabolic engineering and synthetic biology allow for engineering of microbes metabolizing a variety of chemicals including gaseous feeds into a number of biorenewables and transportation liquid fuels. New computational tools enable the systematic exploration of all feasible conversion alternatives. Here we computationally assessed all thermodynamically feasible ways of co-utilizing CH_4_, CO, and CO_2_ using ferric as terminal electron acceptor for the production of all key precursor metabolites. We identified the thermodynamically feasible co-utilization ratio ranges of CH_4_, CO, and CO_2_ toward production of the target metabolite(s) as a function of ferric uptake. A revised version of the iMAC868 genome-scale metabolic model of *Methanosarcina acetivorans* was chosen to assess co-utilization of CH_4_, CO, and CO_2_ and their conversion into selected target products using the optStoic pathway design tool. This revised version contains the latest information on electron flow mechanisms by the methanogen while supplied with methane as the sole carbon source. The interplay between different gas co-utilization ratios and the energetics of reverse methanogenesis were also analyzed using the same metabolic model.

## Introduction

The global increase in oil production, fossil fuel combustion, biomass burning, and hydraulic fracturing of shale gas and climate change concerns has motivated the reduction of emissions from anthropogenic sources. Mitigation of gaseous emissions (such as methane, carbon dioxide, and carbon monoxide) from the environment and their microbial conversion into useful products provides a sustainable and transformative solution that avoids the “food vs. fuel” dilemma. Methane, the major constituent of natural gas, has the highest oxidation potential amongst carbon dioxide, carbon monoxide, and glucose to be converted into a wide range of products including liquid fuels such as ethanol and butanol. Carbon monoxide, often as synthesis gas with varying levels of carbon dioxide and hydrogen (Aasberg-Petersen et al., 2001), along with methane could yield a variable mixture of gases that can be tapped for microbial conversion.

Existing chemical gas-to-liquid (GTL) technologies (i.e., GTL process using the Fischer-Tropsch method) require high operating temperatures and pressures, involve high CapEx costs, yield generally low carbon conversion efficiency, and cannot directly convert methane into the desired bioproducts (Dry, 2002; Steynberg, 2004; Haynes and Gonzalez, 2014). The biological routes of methane utilization, have received renewed interest because of process simplicity (Lopez et al., 2013), selectivity toward targeted pathways (Haynes and Gonzalez, 2014; Mueller et al., 2015), and recent advancements in the characterization and genetic tools of methanotrophic microbes enabling direct transformation of methane into valuable chemicals and fuel molecules (Coleman et al., 2014; Fei et al., 2014; Strong et al., 2015; Henard et al., 2016). Much of the current industrial applications of methane utilization have been devoted to the use of aerobic methanotrophic bacteria (Fei et al., 2014). In contrast, the global methane cycle is primarily controlled by the syntrophy of microorganisms living in anoxic environments. Although biological methane conversion can occur in oxic habitats (Conrad, 2009; Knittel and Boetius, 2009), more than 80% of methane produced in the world's oceans is estimated to be converted anaerobically (Orphan et al., 2001). In addition, anaerobic routes for methane metabolism offer better carbon and energy efficiency compared with aerobic pathways (Mueller et al., 2015; Nazem-Bokaee et al., 2016). Difficulties in culturing anaerobic methanotrophs in the lab, arising from syntrophy requirements, have hampered their rapid characterization and application. Nonetheless, recent observation of methane utilization by anaerobic methanotrophic archaea (ANME) decoupled from their sulfate-reducing bacteria (SRB) partners in the presence of artificial electron acceptors (Scheller et al., 2016) revealed new avenues for direct anaerobic conversion of methane by ANMEs into useful chemicals. So far there is no microbe capable of AOM utilizing other gaseous substrates at industrial scale. Acetogens has been the workhouse for gas fermentation in industry for over two decades. Anaerobic conversion of carbon monoxide into valuable products such as ethanol, acetate, and 2,3-butanediol at industrial scale has been pursued using different strains of Clostridium (Simpson et al., 2010; Köpke et al., 2011a,b; Tran and Simpson, 2015; Daniell et al., 2016; Martin et al., 2016). A recent study on the co-utilization of carbon dioxide and carbon monoxide or hydrogen to produce acetate using *Moorella thermoacetica* (Hu et al., 2016) further demonstrates the need for systematic study of co-utilization of various C_1_ gases in other potential microbial hosts.

In this work, we aim at developing a computational framework allowing for designing overall thermodynamically feasible conversions of mixes of gaseous molecules into selected metabolites and, then, investigating the metabolic capabilities of a selected microorganism in response to introducing new gas mixture combinations. Using optStoic (Chowdhury and Maranas, 2015) we exhaustively identified all thermodynamically feasible optimal conversion stoichiometries making use of a combination of CH_4_, CO, and CO_2_. Note that there exist many other computational tools for pathway design (Hadadi and Hatzimanikatis, 2015; Long et al., 2015; Nazem-Bokaee and Senger, 2015; Huang et al., 2017). Ten key branch point (precursor) metabolites (Noor et al., 2010) were selected owing to their essentiality for anabolic processes found in all forms of life as well as their crucial role as building blocks for producing many commodity and specialty chemicals listed as top value-added chemicals by the U.S. Department Of Energy (DOE). Maximum uptake of carbon coming from CH_4_, CO, or CO_2_ and their co-utilization ratios have been assessed as well as the indispensability of ferric ion as an electron acceptor. To analyze metabolic pathway usage at different co-utilization ratios of CH_4_, CO, and CO_2_ designed by optStoic algorithm, a revised version of the iMAC868 genome-scale metabolic model of the methanogenic archaeon *Methanosarcina acetivorans* (Nazem-Bokaee et al., 2016) was used allowing for full tracking of carbon and electron flow within the reversal of methanogenesis pathway. Recent studies identified the existence of an electron bifurcating multi-complex enzyme, cytosolic heterodisulfide reductase HdrABC, shedding light into pathways for utilizing methane by *M. acetivorans* in the presence of ferric to produce useful chemicals such as acetate (Yan et al., 2017; Nazem-Bokaee et al., 2018). It has been shown before that *M. acetivorans* is capable of growing with carbon monoxide (Rother and Metcalf, 2004; Lessner et al., 2006) and metabolizing carbon dioxide (in the form of bicarbonate) along with methane (Soo et al., 2016), thus, making the archaeon a suitable platform to study the conversion of varying mixtures of these gases into useful products. The computational framework put forth in this study could inform design of novel metabolic engineering strategies for the industrial production of bio-based chemicals and liquid fuels from mixed gaseous feeds.

## Methods

### Computational design of overall stoichiometries for gas co-utilization

To explore optimal overall stoichiometries for conversion of gaseous molecules (i.e., CH_4_, CO, and CO_2_) into target products, the optStoic procedure (Chowdhury and Maranas, 2015) was implemented in Python so that it can be freely accessible (Supplementary Data Sheet 2). The goal was to design overall stoichiometries informing thermodynamically feasible co-utilization of the gaseous molecules leading to the production of 10 C-mol of products listed in Table 1 (equation 1).


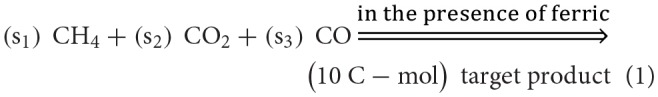


In the postulated overall stoichiometry s_1_, s_2_, and s_3_ are the optimal coefficients of methane, carbon dioxide, and carbon monoxide, respectively. Because the target products listed in Table 1 contain varying number of carbons, fixing the stoichiometry of target product in Equation 1 enables a direct comparison of gaseous feed ratios on a per carbon mol basis. In the optStoic algorithm, water molecules and protons can be taken up or produced as needed so that Equation 1 remains elementally and charge balanced. Furthermore, phosphate, ammonia, and hydrogen sulfide were added to balance Equation 1 when a target product contains phosphorous, nitrogen, and sulfur, respectively. No carbon-containing compound other than methane, carbon dioxide, and carbon monoxide was allowed as an additional substrate. The choice of the products listed in Table 1 is based on their essentiality in the metabolism of almost all forms of life (Noor et al., 2010) and their significance in being used as building blocks of many commodity and specialty chemicals as mentioned in the DOE list of top value-added chemicals. The performance criteria of the overall conversion shown in Equation 1 were to maximize s_1_, s_2_, or s_3_ separately at a specified ferric uptake. To safeguard the thermodynamic feasibility of all conversions, the minimum overall standard ΔG was set to be less than zero. A previously assembled database of metabolites (Chowdhury and Maranas, 2015) was used to explore the optimal combination of reactants and products for any given overall stoichiometry. COBRApy (Ebrahim et al., 2013) with built-in cGLPK (http://www.gnu.org/software/glpk/) solver was used to solve the optimization problems written in Python 2.7.

**Table 1:** The key branch point (precursor) metabolites essential for anabolic processes found in all forms of life considered as target products of gaseous fermentation in this study.

**Target product**	**Chemical formula**	**Degree of reduction**
Pyruvate (**PYR**)	C_3_H_3_${\text{O}}_{3}^{-}$	3
Phosphoenolpyruvate (**PEP**)	C_3_H_3_O_6_P^2−^	2.66
Glyceraldehyde-3-phosphate (**GAP**)	C_3_H_6_O_6_P^−^	3.66
Oxaloacetate (**OXA**)	C_4_H_2_${\text{O}}_{5}^{2-}$	2
Erythrose-4-phosphate (**E4P**)	C_4_H_8_O_7_P^−^	3.75
Ribose-5-phosphate (**R5P**)	C_5_H_10_O_8_P^−^	3.8
2-ketoglutarate (**2KG**)	C_5_H_4_${\text{O}}_{5}^{2-}$	2.8
Glucose-6-phosphate (**G6P**)	C_6_H_12_O_9_P^−^	3.83
Acetyl CoA (**ACA**)	C_23_H_35_O_17_N_7_P_3_S^3−^	4.04
Succinyl-CoA (**SCA**)	C_25_H_36_O_19_N_7_P_3_S^4−^	3.92

### Modifications to the iMAC868 metabolic model of *M. acetivorans*

**Figure 1:**
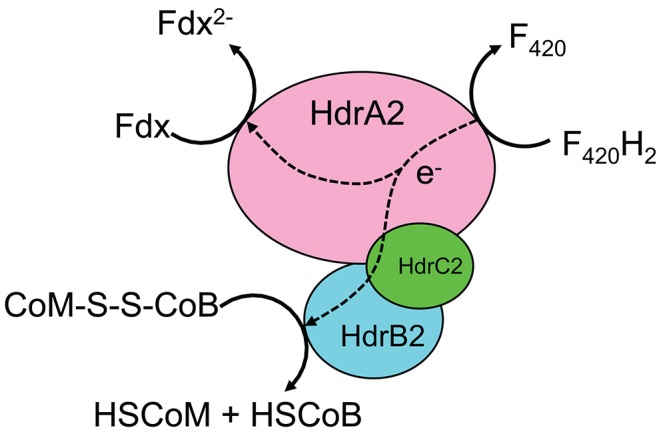
Electron bifurcation mechanism by HdrA2B2C2 complex of *M. acetivorans* in the presence of external electron acceptor when grown with methane (see Yan et al., 2017 for more details). F_420_: Cofactor F_420_; F_420_H_2_: reduced form of cofactor F_420_; Fdx: ferredoxin; Fdx^2−^: reduced form of ferredoxin; HSCoM: coenzyme M; HSCoB: coenzyme B; CoM-S-S-CoB: heterodisulfide.

Since the development and release of the iMAC868 metabolic model (Nazem-Bokaee et al., 2016), there have been new experimental studies aimed at better understanding the electron flow mechanisms and biochemistry of *M. acetivorans* growing on methane (Yan et al., 2017, 2018; Nazem-Bokaee et al., 2018). This provided the impetus for updating the iMAC868 model of this methanogen to catalog these findings. It was recently shown that *M. acetivorans* expresses a multi-unit cytosolic heterodisulfide reductase complex, HdrA2B2C2, when grown with methane in the presence of ferric (Yan et al., 2017; Yan and Ferry, 2018) that can partition electrons (i.e., bifurcate electrons) coming from cofactor F_420_ (reduced) between ferredoxin (with lower electrode potential) and heterodisulfide (with higher electrode potential) (Figure 1). Therefore, HdrA2B2C2 complex bypasses thermodynamic uphill for direct electron transfer from cofactor F_420_ to ferredoxin during ferric-dependent methanotrophy by *M. acetivorans*. This important finding introduces a new metabolic capability of *M. acetivorans* and, therefore, was cataloged in the updated version of the iMAC868 metabolic model. The resulting coenzyme M and coenzyme B are re-used to regenerate heterodisulfide used for activation of methane. The reduced ferredoxin is used to drive the biosynthesis of acetyl-CoA by CO dehydrogenase, Cdh. Therefore, we replaced the previously used electron flow routes in our model with the new route representing the newly elucidated function of HdrA2B2C2 (see Figure 1). We found that the model accommodated the new electron bifurcation mechanism providing new insights about the key role of ferric in the distribution of electrons between major products of methanotrophy as well as on energy conservation mechanisms (Nazem-Bokaee et al., 2018). The model was assembled in a format compatible for flux balance analysis (Orth et al., 2010). FBA optimization problems were solved by GNU Linear Programming Kit (GLPK) (http://www.gnu.org/software/glpk/) solver in Matlab using COBRA toolbox (Schellenberger et al., 2011). Flux variability analysis (FVA) was performed to obtain range of fluxes under optimal growth conditions as described previously (Mahadevan and Schilling, 2003). Both FBA and FVA problems incorporated overall thermodynamic feasibility constraints (overall ΔG≤0).

## Results and discussion

### Thermodynamically feasible gas co-utilization stoichiometries designed by optstoic

**Figure 2:**
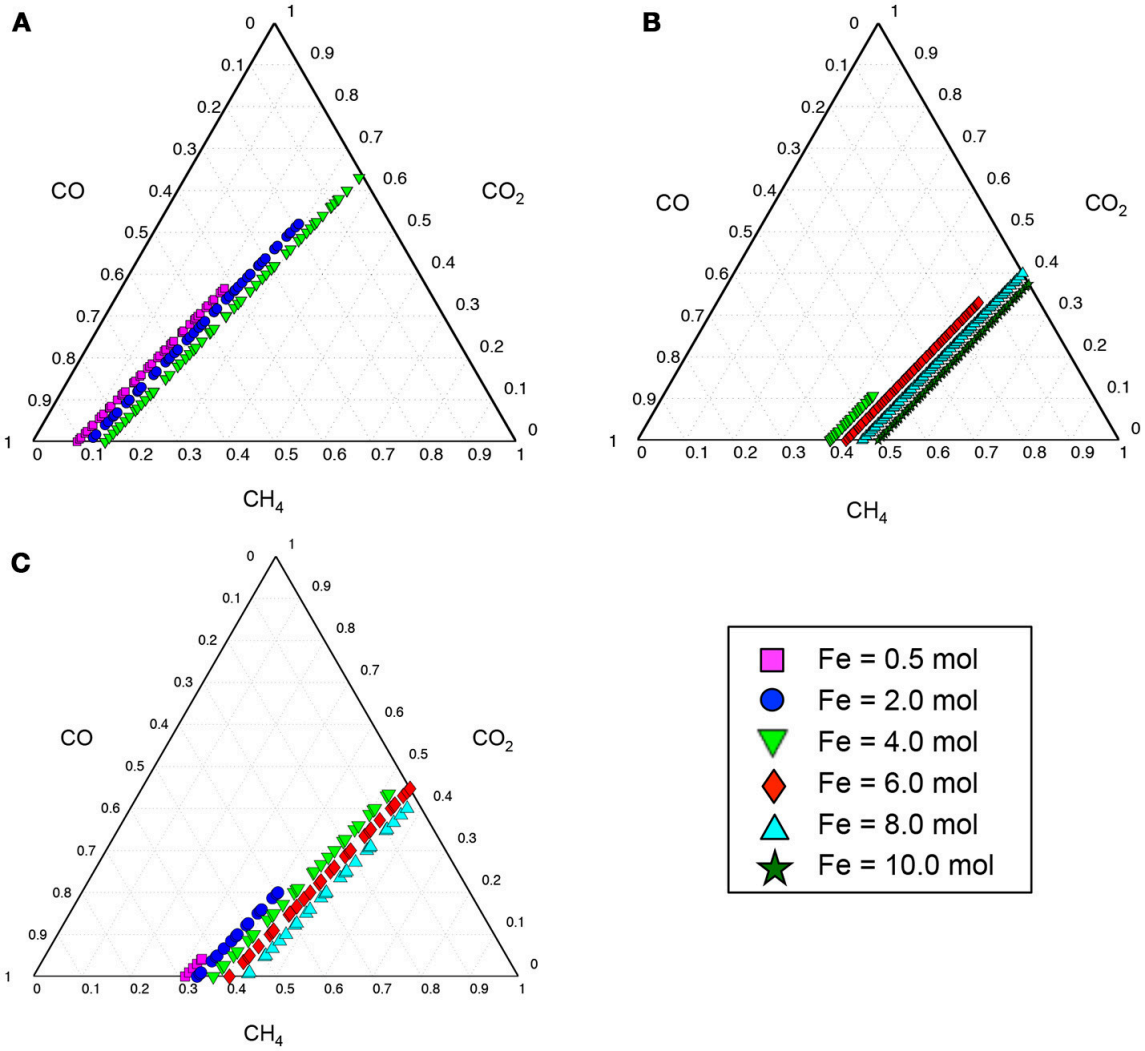
Ternary diagrams showing the contribution of gaseous carbon sources (i.e., CH_4_, CO, or CO_2_) in the production of 10 C-mol oxaloacetate **(A)**, glyceraldehyde-3-phosphate **(B)**, or acetyl-CoA **(C)** as selected target products. Colorful symbols on the bottom right of the figure show the range of ferric (Fe^3+^) uptake (in moles) at which the overall gases-to-product conversion shown in Equation 1 is thermodynamically feasible. Each symbol on the ternary plots represents a single independent thermodynamically feasible stoichiometric conversion of gases-to-product simulated by optStoic algorithm. In each simulation, the stoichiometries of the target product, ferric, and one of the gases are fixed and the objective is to maximize the stoichiometries of the other two gases. The moles of CH_4_, CO, or CO_2_ in the overall stoichiometry are normalized to be between zero and one in the ternary diagram.

The thermodynamically feasible ranges of co-utilization of CH_4_, CO, and CO_2_ for the production of target chemicals listed in Table 1 were predicted by optStoic to be dependent on the level of available ferric. Figure 2 shows this dependency for three selected products with varying degrees of reduction. As the ferric level goes up (i.e., increasing the electron sink capacity), methane usage increases in proportion. However, only some ratio ranges of the CH_4_-CO-CO_2_ triplet lead to thermodynamically feasible production of the target molecules (Figure 2). Here, increasing ferric levels provides opportunity for CO_2_ utilization levels to go up by accepting electrons coming from methane. This increase, however, is at the expense of reduction in CO utilization levels to satisfy stoichiometric and thermodynamics feasibility of the overall conversion.

The minimum and maximum moles of ferric required to maintain any thermodynamically feasible gas co-utilization are given in Table 2 for all target products listed in Table 1. For example, a minimum of 3.04 mol ferric was required to obtain any feasible conversion of gaseous substrates toward glyceraldehyde-3-phosphate (GAP) while no feasible overall stoichiometry was found with a methane carbon contribution <38%. The overall conversions given in Table 2 also unmask the possibility of designing gas bi-utilization (where either CH_4_ and CO or CH_4_ and CO_2_ can be co-utilized) at varying levels of ferric, which is further explained in the following sections.

**Table 2:** optStoic-predicted overall stoichiometric conversions (middle column) for which the stoichiometry of CH_4_, CO_2_, or CO were maximized independently.

**Optimization conditions**	**Overall stoichiometries**	**ΔG (kcal)**
Pyruvate (**PYR**)		
*max*. *s*_*C*_*H*__4__	$4.999\;CH_{4}+5.000\;CO_{2}+0.0015\;H_{2}O+6.66\;Fe^{3+}$ $\to {3.333\;C}_{3}H_{3}O_{3}^{-}+9.993\;H^{+}+6.66\;Fe^{2+}$	−87
*max*. *s*_*C*_*O*__2__	$4.603\;CH_{4}+5.395\;CO_{2}+3.5\;Fe^{3+}$ $\to {3.33\;C}_{3}H_{3}O_{3}^{-}+0.791\;H_{2}O+6.833\;H^{+}+3.5\;Fe^{2+}$	−12
*max*. *s*_*CO*_	$2.222\;CH_{4}+7.777\;CO+2.222\;H_{2}O\to {3.333\;C}_{3}H_{3}O_{3}^{-}+3.333\;H^{+}$	−40
Phosphoenolpyruvate (**PEP**)		
*max*. *s*_*C*_*H*__4__	$5.416\;CH_{4}+4.583\;CO_{2}+3.333\;HPO_{4}^{2-}+10\;Fe^{3+}$ $\to {3.333\;C}_{3}H_{3}O_{6}P^{2-}+2.499\;H_{2}O+10\;H^{+}+10\;Fe^{2+}$	−128
*max*. *s*_*C*_*O*__2__	$4.791\;CH_{4}+5.208\;CO_{2}+3.333\;HPO_{4}^{2-}+5\;Fe^{3+}$ $\to {3.333\;C}_{3}H_{3}O_{6}P^{2-}+3.749\;H_{2}O+5\;H^{+}+5\;Fe^{2+}$	−16
*max*. *s*_*CO*_	$2.245\;CH_{4}+7.754\;CO+3.333\;HPO_{4}^{2-}+0.14\;Fe^{3+}$ $\to {3.333\;C}_{3}H_{3}O_{6}P^{2-}+1.087\;H_{2}O+0.14\;H^{+}+0.14\;Fe^{2+}$	−5
Glyceraldehyde 3-phosphate **(GAP)**		
*max*. *s*_*C*_*H*__4__	$6.249\;CH_{4}+3.750\;CO_{2}+3.333\;HPO_{4}^{2-}+10\;Fe^{3+}$ $\to {3.333\;C}_{3}H_{6}O_{6}P^{-}+0.833\;H_{2}O+6.667\;H^{+}+10\;Fe^{2+}$	−82
*max*. *s*_*C*_*O*__2__	$5.874\;CH_{4}+4.124\;CO_{2}+3.333\;HPO_{4}^{2-}+7\;Fe^{3+}$ $\to {3.333\;C}_{3}H_{6}O_{6}P^{-}+1.583\;H_{2}O+3.667\;H^{+}+7\;Fe^{2+}$	−11
*max*. *s*_*CO*_	$3.840\;CH_{4}+6.159\;CO+0.506\;H_{2}O+3.333\;HPO_{4}^{2-}+0.293\;H^{+}+3.04\;Fe^{3+}$ $\to {3.333\;C}_{3}H_{6}O_{6}P^{-}+3.04\;Fe^{2+}$	−5
Oxaloacetate (**OXA**)		
*max*. *s*_*C*_*H*__4__	$3.75\;CH_{4}+6.25\;CO_{2}+5\;Fe^{3+}\to {2.5\;C}_{4}H_{2}O_{5}^{-2}+10\;H^{+}+5\;Fe^{2+}$	−53
*max*. *s*_*C*_*O*__2__	$3.50\;CH_{4}+6.50\;CO_{2}+3\;Fe^{3+}\to {2.5\;C}_{4}H_{2}O_{5}^{-2}+0.50\;H_{2}O+{8\;H}^{+}+3\;Fe^{2+}$	−6
*max*. *s*_*CO*_	$0.83\;CH_{4}+9.17\;CO+3.33\;H_{2}O\to {2.5\;C}_{4}H_{2}O_{5}^{-2}+5\;H^{+}$	−65
Erythrose-4-phosphate (**E4P**)		
*max*. *s*_*C*_*H*__4__	$6.25\;CH_{4}+3.75\;CO_{2}+2.50\;HPO_{4}^{2-}+10\;Fe^{3+}$ $\to {2.50\;C}_{4}H_{8}O_{7}P^{-}+7.50\;H^{+}+10\;Fe^{2+}$	−86
*max*. *s*_*C*_*O*__2__	$5.875\;CH_{4}+4.125\;CO_{2}+2.50\;HPO_{4}^{2-}+7\;Fe^{3+}$ $\to {2.50\;C}_{4}H_{8}O_{7}P^{-}+0.75\;H_{2}O+4.5\;H^{+}+7\;Fe^{2+}$	−15
*max*. *s*_*CO*_	$3.81\;CH_{4}+6.19\;CO+1.31\;H_{2}O+2.50\;HPO_{4}^{2-}+2.85\;Fe^{3+}$ $\to {2.50\;C}_{4}H_{8}O_{7}P^{-}+0.35\;H^{+}+2.85\;Fe^{2+}$	−5
Ribose-5-phosphate (**R5P**)		
*max*. *s*_*C*_*H*__4__	$6.25\;CH_{4}+3.75\;CO_{2}+0.5\;H_{2}O+2\;HPO_{4}^{2-}+10\;Fe^{3+}$ $\to {2\;C}_{5}H_{10}O_{8}P^{-}+8\;H^{+}+10\;Fe^{2+}$	−92
*max*. *s*_*C*_*O*__2__	$5.81\;CH_{4}+4.19\;CO_{2}+2\;HPO_{4}^{2-}+6.5\;Fe^{3+}$ $\to {2\;C}_{5}H_{10}O_{8}P^{-}+0.375\;H_{2}O+4.5\;H^{+}+6.5\;Fe^{2+}$	−10
*max*. *s*_*CO*_	$3.76\;CH_{4}+6.24\;CO+1.75\;H_{2}O+2\;HPO_{4}^{2-}+2.55\;Fe^{3+}$ $\to {2\;C}_{5}H_{10}O_{8}P^{-}+0.55\;H^{+}+2.55\;Fe^{2+}$	−5
2-ketoglutarate (**2KG**)		
*max*. *s*_*C*_*H*__4__	$4.75\;CH_{4}+5.25\;CO_{2}+6\;Fe^{3+}$ $\to {2\;C}_{5}H_{4}O_{5}^{2-}+0.50\;H_{2}O+10\;H^{+}+6\;Fe^{2+}$	−85
*max*. *s*_*C*_*O*__2__	$4.38\;CH_{4}+5.\;62\;CO_{2}+3\;Fe^{3+}$ $\to {2\;C}_{5}H_{4}O_{5}^{2-}+1.25\;H_{2}O+7\;H^{+}+3\;Fe^{2+}$	−14
*max*. *s*_*CO*_	$2\;CH_{4}+8\;CO+2\;H_{2}O\to {2\;C}_{5}H_{4}O_{5}^{2-}+4\;H^{+}$	−57
Glucose-6-phosphate (**G6P**) and Fructose-6-phosphate (**F6P**)		
*max*. *s*_*C*_*H*__4__	$6.248\;CH_{4}+3.748\;CO_{2}+1.666\;HPO_{4}^{2-}+0.834\;H_{2}O+10\;Fe^{3+}$ $\to {1.666\;C}_{6}H_{12}O_{9}P^{-}+8.334\;H^{+}+10\;Fe^{2+}$	−188
*max*. *s*_*C*_*O*__2__	$5.811\;CH_{4}+4.185\;CO_{2}+1.666\;HPO_{4}^{2-}+6.5\;Fe^{3+}$ $\to {1.666\;C}_{6}H_{12}O_{9}P^{-}+0.041\;H_{2}O+{4.834\;H}^{+}+6.5\;Fe^{2+}$	−11
*max*. *s*_*CO*_	$3.744\;CH_{4}+6.252\;CO+2.078\;H_{2}O+1.666\;HPO_{4}^{2-}+2.474\;Fe^{3+}$ $\to {1.666\;C}_{6}H_{12}O_{9}P^{-}+{0.808\;H}^{+}+2.474\;Fe^{2+}$	−5
Acetyl-CoA (**ACA**)		
*max*. *s*_*C*_*H*__4__	$5.817\;CH_{4}+4.187\;CO_{2}+1.305\;HPO_{4}^{2-}+{3.045\;NH}_{3}+{0.435\;H}_{2}S+8.26\;Fe^{3+}$ $\to {0.435\;C}_{23}H_{35}O_{17}{N_{7}P_{3}S}^{3-}+10\;H^{+}+6.2\;H_{2}O+8.26\;Fe^{2+}$	−99
*max*. *s*_*C*_*O*__2__	$5.410\;CH_{4}+4.595\;CO_{2}+1.305\;HPO_{4}^{2-}+{3.045\;NH}_{3}+{0.435\;H}_{2}S+5\;Fe^{3+}$ $\to {0.435\;C}_{23}H_{35}O_{17}{N_{7}P_{3}S}^{3-}+7.015\;H_{2}O+6.74\;H^{+}+5\;Fe^{2+}$	−22
*max*. *s*_*CO*_	$3.064\;CH_{4}+6.941\;CO+1.305\;HPO_{4}^{2-}+{3.045\;NH}_{3}+{0.435\;H}_{2}S+0.117\;Fe^{3+}$ $\to {0.435\;C}_{23}H_{35}O_{17}{N_{7}P_{3}S}^{3-}+4.765\;H_{2}O+1.857\;H^{+}+0.117\;Fe^{2+}$	−5
Succinyl-CoA (**ACA**)		
*max*. *s*_*C*_*H*__4__	$5.7\;CH_{4}+4.3\;CO_{2}+1.2\;HPO_{4}^{2-}+{2.8\;NH}_{3}+{0.4\;H}_{2}S+8\;Fe^{3+}$ $\to {0.4\;C}_{25}H_{36}O_{19}{N_{7}P_{3}S}^{4-}+10\;H^{+}+5.8\;H_{2}O+8\;Fe^{2+}$	−97
*max*. *s*_*C*_*O*__2__	$5.262\;CH_{4}+4.738\;CO_{2}+1.2\;HPO_{4}^{2-}+{2.8\;NH}_{3}+{0.4\;H}_{2}S+4.5\;Fe^{3+}$ $\to {0.4\;C}_{25}H_{36}O_{19}{N_{7}P_{3}S}^{4-}+6.675\;H_{2}O+6.5\;H^{+}+4.5\;Fe^{2+}$	−15
*max*. *s*_*CO*_	$2.933\;CH_{4}+7.066\;CO+1.2\;HPO_{4}^{2-}+{2.8\;NH}_{3}+{0.4\;H}_{2}S$ $\to {0.4\;C}_{25}H_{36}O_{19}{N_{7}P_{3}S}^{4-}+4.266\;H_{2}O+2\;H^{+}$	−9

*Bold numbers are the maximum feasible stoichiometry of a gas molecule under the optimization conditions shown on the left column. The stoichiometry of the target product only was fixed to moles equivalent to 10 C-mol carbon. ΔG of formation of the overall conversions were also given (right column)*.

**Figure 3:**
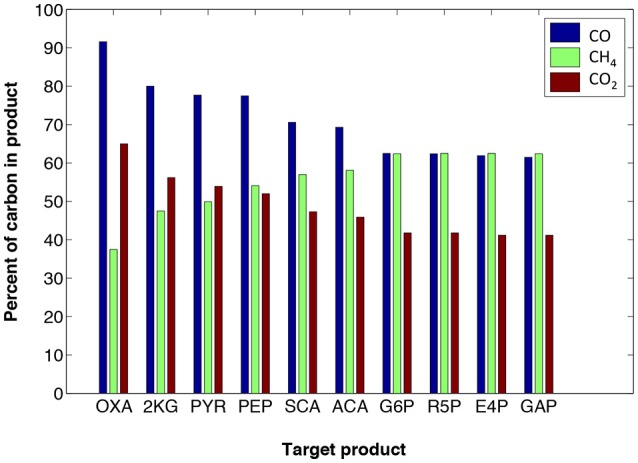
optStoic-predicted maximum carbon (shown as percentage on the Y-axis) contribution from carbon monoxide (blue), methane (green), or carbon dioxide (red) for the production of different target products (For abbreviations see Table 1). These maxima are from different independent overall stoichiometry designs predicted by optStoic (see Table 2 for all stoichiometries and performance criteria).

The maximum amount of carbon that can be incorporated to target products from CH_4_, CO, or CO_2_, depends on the target molecule C/O ratio and reduction level. Figure 3 displays how the choice of target molecule (those listed in Table 1) affects the maximum carbon contributed by the three gaseous feeds. For example, CO could be the top supplier of carbon for oxaloacetate (OXA), as expected, because OXA is highly oxidized. Note that Figure 3 does not directly represent maximum co-utilization of ratios of gases; however, it demarcates the theoretical limits on utilizing any of the gases for the production of each target product. For example, under the defined criteria for optStoic, it would be thermodynamically infeasible to design an overall stoichiometry for pyruvate production in which carbon coming from methane co-utilized with other gases exceeded 50% (see also Table 2 for all stoichiometric designs).

Nonetheless, methane contributes the most carbon at maximum ferric uptake levels. In addition, imposing a more negative requirement for the overall standard free energy of change results in less carbon contributed from CO_2_ (since it has the lowest Gibbs free energy of formation among CO and CH_4_) leading to a decline in maximum co-utilization ratios of CO_2_-to-CH_4_. Such information could be useful in designing and/or modifying bioconversions based on varying compositions of industrial gas waste streams (Subramani and Gangwal, 2008; Lackey et al., 2015).

In the following section we describe how overall stoichiometry designs generated by optStoic could be used to inform metabolic engineering strategies through using the updated iMAC868 metabolic model of *M. acetivorans* as a platform.

### Metabolic capabilities of *M. acetivorans* during gas co-utilization

The ratio of industrial waste gases is often highly variable from stream to stream leading to difficulties in predicting desirable gas stream-to-target product conversions (Williams et al., 2007; Subramani and Gangwal, 2008). The optStoic designs could serve as a guide to estimate feasible conversions using the metabolic model of *M. activorans*. We selected oxaloacetate (OXA), glyceraldehyde 3-phosphate (GAP), and acetyl-CoA (ACA) (out of hundreds of unique overall stoichiometry designs) based on their distinct differences as shown in Figures 2, 3 (also in Table 1) as well as their importance as building blocks of numerous valuable end products. We chose the stoichiometric ratios of CH_4_, CO, and CO_2_ at an arbitrary ferric level of 4 moles at which co-utilization of the three gases for the production of OXA, ACA, and GAP was predicted by optStoic to be thermodynamically feasible (Table 3). To implement these stoichiometric ratios in the context of the metabolic model of *M. acetivorans*, the lower and upper bounds of the reactions corresponding to the uptake of CH_4_, CO, and CO_2_ in the iMAC868 metabolic model were fixed to the stoichiometric ratios of CH_4_, CO, and CO_2_ shown in Table 3. Analysis of the flux distribution through the metabolic network confirmed the usage of the reversal of the methanogenesis pathway indicating the incorporation of the gaseous substrates into biomass and cofactor biosynthesis. The iMAC868 metabolic model also predicts the uptake of ammonia, hydrogen sulfide, and phosphate as essential sources of nitrogen, sulfur, and phosphorus, respectively, consistent with the overall optStoic design. Flux variability analysis results in predicting a maximum yield of 2.499 (mol per 10 C-mol of gases) for OXA. This is in agreement with a 2.5 stoichiometric value predicted by optStoic leading to the same ratio of CH_4_, CO, and CO_2_ co-utilization implying that metabolism remains unaffected even at maximum OXA production yield. The maximum yields of GAP and ACA predicted by the iMAC868 metabolic model are 2.944 and 0.388 (mol per 10 C-mol of gases), respectively, which is only 11.5, and 11% less than the optimal overall stoichiometries obtained by optStoic. This difference is due to the inclusion of many more cofactors and intermediate metabolites in the metabolic network compared to the consideration of one simple overall stoichiometry as that shown in Equation 1. Further analysis of the flux through the formation of biomass, as another product of the metabolic network, reveals a maximum biomass yield of 0.217 at a ferric level of 4.2 (mol per 10 C-mol of gases) when using the gas ratios optimized for ACA production predicted by optStoic (see Table 3). Therefore, the optStoic design could quickly inform potential gas co-utilization ratios at which a certain level of cellular growth can be achieved. It should be noted that there exist other possible gas co-utilization ratios that could end up obtaining similar biomass yields. For example, using the gas ratios optimized for GAP production (see Table 3) results in achieving a maximum biomass yield of 0.224 at a ferric level of 4.5 (mol per 10 C-mol of gases), which is only 3% higher than what could be achieved at a gas composition optimized for ACA production and is slightly richer in CO (see Table 3).

**Table 3:** optStoic-designed stoichiometries (mol) of methane (*s*_*CH*_4__), carbon monoxide (*s*_*CO*_), and carbon dioxide (*s*_*CO*_2__) resulted in the production of 10 C-mol of three selected target products used to constrain the *in silico* uptake of these gases by the iMAC868 metabolic model of *M. acetivorans*.

**Target Product**	**Gas Composition**		
	**s_CH_4__**	**s_CO_**	**s_CO_2__**
Glyceraldehyde-3-phosphate (GAP)	4.333	4.666	1
Oxaloacetate (OXA)	1.833	7.166	1
Acetyl-CoA (ACA)	4.04	4.96	1

It has been postulated that *M. acetivorans* reduces ferric at multi-heme c-type cytochromes sites to which electrons are shuttled by membrane-bound methanophenazine (Yan et al., 2017). Depending on the composition of gaseous substrates being used (given in Table 3), the iMAC868 metabolic model predicts that at least 13% (up to 20%) of heterodisulfide has to be reduced through the membrane-bound heterodisulfide reductase (HdrDE) that reduces methanophenazine. The remaining heterodisulfide can be reduced via either the cytosolic HdrA2B2C2 or HdrDE. Reduced cofactor F_420_, which donates electrons to ferredoxin and heterodisulfide at the HdrA2 site, can be regenerated through any of F_420_ dehydrogenase (Fpo), F_420_-dependent methylene-H_4_MPT reductase (Mer), F_420_-dependent methylene-H_4_MPT dehydrogenase (Mtd), or F_420_-dependent NADP reductase enzyme complexes according to the metabolic model predictions.

**Figure 4:**
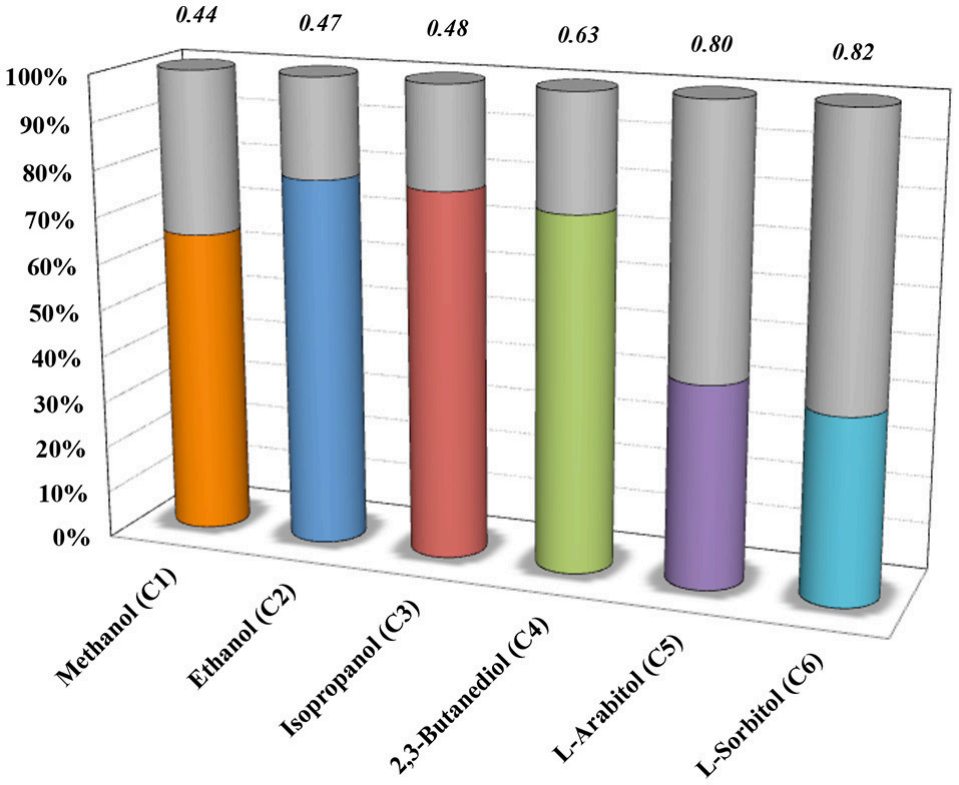
optStoic-predicted co-production of selected alcohols (with their number of carbons given in parenthesis) along with butanol in the presence of ferric as electron acceptor. Y-axis indicates that under the design criteria of optStoic, where the only products of CO and CH_4_ co-utilization are butanol and one of the shown alcohols, how much (in percent) of the total product could be each alcohol molecule (gray area of the bars show percent butanol of the total). Italic numbers on top of the bars show CO to CH_4_ gas co-utilization ratios.

To further explore the metabolic capabilities of *M. acetivorans*, we decided to analyze the theoretical limits of ethanol and butanol co-production during CH_4_ and CO co-utilization by the iMAC868 model. The biological co-production of alcohols has been reported in the literature where acetone/butanol/ethanol (ABE) fermentation process by clostridial strains has been studied the most and implemented industrially (Worden et al., 1991; Lee et al., 2008; Tracy et al., 2012; Carlson and Papoutsakis, 2017; Fernandez-Naveira et al., 2017). However, most traditional ABE process suffers from high feedstock costs (Green, 2011) and, thus, the use of cheap sources such as C_1_ gas substrates suggests a promising alternate route (Dürre, 2017). Nonetheless, the current C_1_ gas fermentation technology is mainly relied on making use of acetogens (De Tissera et al., 2017). Here, the motivation was to study the co-production of alcohols from co-utilization of C_1_ gaseous substrates in non-traditional hosts such as *M. acetivorans*. For that, first, optStoic was used to design overall conversions such that CH_4_ and CO co-utilization (using one mole of ferric as basis) results in production of one mole butanol while maximizing the production of ethanol and several other alcohol molecules as co-products (Figure 4). optStoic was also applied to examine how conversion of CH_4_ and CO to butanol would vary for different electron acceptors other than ferric. Almost all electron acceptors examined allowed for the same ratio of CH_4_ and CO co-utilization except for thrithionate that enabled about three times higher co-utilization ratio (Supplementary Figure S1 in Data Sheet 1). However, ethanol production as a co-product of butanol production when using trithionate/bisulfate as the electron acceptor pair was only 0.3% of that achievable by using ferric/ferrous as electron acceptor pair. Thus, the overall conversion design using ferric as electron acceptor was employed for analyzing metabolic capabilities of *M. acetivorans* for co-production of ethanol and butanol. The original version of the iMAC868 metabolic model comprises the biosynthetic pathways for ethanol and butanol production (Nazem-Bokaee et al., 2016).

**Figure 5:**
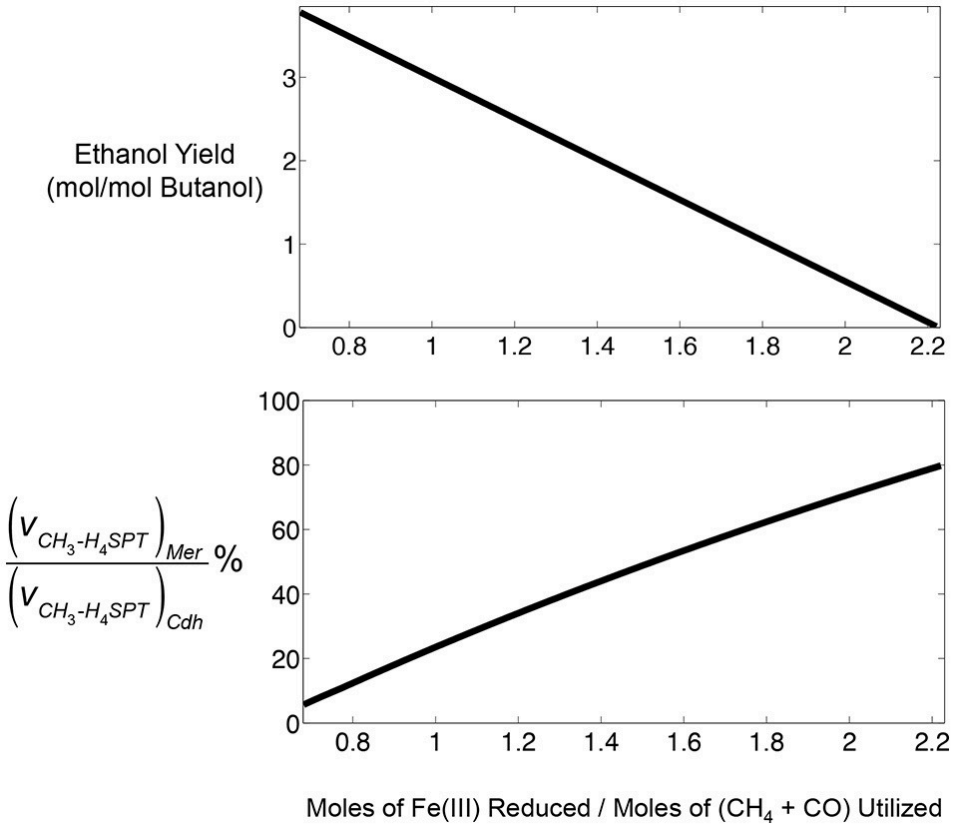
Predictive capabilities of iMAC868 metabolic model of *M. acetivorans* during CO and CH_4_ co-utilization in the presence of ferric for butanol and ethanol co-production. **Top panel**: prediction of ethanol and butanol co-production feasibility over a range of ferric reduction levels. **Bottom panel**: partitioning of methyl-tetrahydrosarcinapterin (CH_3_-H_4_SPT) flux (denoted as *v*) between CO_2_ pathway (Mer) and acetyl-CoA biosynthesis pathway (Cdh) during reversal of the methanogenesis pathway by *M. acetivorans*.

By constraining the lower and upper bounds of the reaction corresponding to the exchange of butanol in the metabolic model to one, and fixing the bounds of reactions corresponding to the uptake of CH_4_ and CO to the respective ratio given in Figure 4 (i.e., 0.47), the model predicts that a maximum of 3.779 moles of ethanol per mole of butanol could be produced (Figure 5). The ethanol-to-butanol molar ratio predicted by optStoic at the same gas co-utilization ratio was 3.682, which is only 2.5% different from that predicted by the iMAC868 metabolic model.

Ethanol co-production with butanol was predicted by iMAC868 metabolic model to be feasible over a range of ferric reduction values from 0.68 up to 2.2 (Figure 5). However, ethanol co-production decreases as ferric reduction levels increases because the reducing power for generating acetyl-CoA, the precursor for both ethanol and butanol production, diminishes. The bottom panel of Figure 5 shows that increasing ferric reduction capacity results in re-routing more methane (through Mer) toward the methyotrophic pathway. Thus, acetyl-CoA production via Cdh remains at stoichiometric limits necessary for satisfying fixed amount of butanol production. Nonetheless, the flux through Cdh could never become zero and at ferric levels of 2.2 mol/mol of gases at least 20% of the CH_3_-H_4_SPT has to be converted to acetyl-CoA to maintain cellular growth. This analysis demonstrate the usefulness of computational tools such as optStoic in guiding metabolic engineering design/analysis for a given bioconversion.

## Summary and conclusion

In this work, we have demonstrated the utility of deploying computational tools such as optStoic along with “genome-scale” metabolic modeling to inform optimal metabolic engineering designs and strategies satisfying an overall desired bioconversion. The optStoic formulation allowed for the exploration of all overall conversions rooting from the co-utilization of low-value C_1_ gaseous feedstocks (i.e., CH_4_, CO_2_, and CO) ending up in the production of precursors used for making high-value biorenewables. We targeted ten key branch point metabolites that have been used extensively as building blocks for the production of many commodity and specialty chemicals such as acetate, terpenoids, and synthetic sugars among others. We showed that the proper choice of an electron acceptor (i.e., ferric) could bypass the thermodynamic barriers for electron flow in the gas-to-chemicals conversions. Furthermore, we showed that there exist well defined gas co-utilization ranges, which are feasible at varying levels of ferric, dependent on the choice of target product. Maximum ferric usage as well as maximum carbon contribution from each of the CH_4_, CO_2_, and CO was analyzed that could lead to new or improved gas co-utilization designs. Using optStoic designs as a guide, metabolic capacities of *M. acetivorans* as the model host was examined owing to its diverse substrate utilization abilities and the progress in its genetic engineering tools. Equipped with latest electron flow mechanisms during growth with methane, the iMAC868 metabolic model of *M. acetivorans* provided information on the partitioning of electrons within the methanogenesis reversal pathway as well as on distribution of carbons coming from co-utilization of mixtures of gases toward selected products. The combined use of optStoic and metabolic modeling presented in this work puts forth an efficient platform for quickly exploring *in silico* the feasibility and limits of various gaseous substrate utilization options.

## Author contributions

HN-B wrote computer scripts, performed the simulations and analyses, designed and generated the figures and tables, and wrote the manuscript. CM supervised and contributed to the design of the study, wrote the manuscript, and critically revised the manuscript. Both authors read and approved the final manuscript.

### Conflict of interest statement

The authors declare that the research was conducted in the absence of any commercial or financial relationships that could be construed as a potential conflict of interest.
