# Supplementary material for: A Prospective Study on the Fermentation Landscape of Gaseous Substrates to Biorenewables Using Methanosarcina acetivorans Metabolic Model
Source: Front Microbiol. 2018 Aug 24;9:1855. doi: 10.3389/fmicb.2018.01855 (PMC6117407; doi:10.3389/fmicb.2018.01855)
Supplement: Supplementary file 1 [file Data_Sheet_1.docx]

**Supplementary Materials**

Figure S1. Co-utilization of CH_4_ and CO for the production of one mole butanol in the presence of various electron acceptors predicted by optStoic algorithm.
