# Supplementary material for: A Prospective Study on the Fermentation Landscape of Gaseous Substrates to Biorenewables Using Methanosarcina acetivorans Metabolic Model
Source: Front Microbiol. 2018 Aug 24;9:1855. doi: 10.3389/fmicb.2018.01855 (PMC6117407; doi:10.3389/fmicb.2018.01855)
Supplement: Supplementary file 2 [file Data_Sheet_2.pdf]

```
# This Python script executes the optStoich formulation in Python 2.7
# Written by Hadi Nazem-Bokaee
# Department of Chemical Engineering
# The Pennsylvania State University
# University Park, Pennsylvania 16802, USA
# Last edited August 4, 2018
# Ref: Nazem-Bokaee H and Maranas CD (2018), "A Prospective Study on the Fermentation Landscape of Gaseous Substrates to Biorenewables Using Methanosarcina acetivorans Metabolic Model." Front. Microbiol. 9:1855


# The optStoic formulation was originally developed by Chowdhury and Maranas
# Ref: Chowdhury, A. and C. D. Maranas (2015), "Designing overall stoichiometric conversions and intervening metabolic reactions." Sci Rep 5: 16009


# The input file is "met_database.mat", which is a Matlab structure formatted to be consistent with the structure of COBRA models
# Ref: Schellenberger, J., R. Que, R. M. Fleming, I. Thiele, J. D. Orth, A. M. Feist, D. C. Zielinski, A. Bordbar, N. E. Lewis, S. Rahmanian, J. Kang, D. R. Hyduke and B. O. Palsson (2011), "Quantitative prediction of cellular metabolism with constraint-based models: the COBRA Toolbox v2.0." Nat Protoc 6(9): 1290-1307


# initiating cobrapy (this includes numpy, scipy etc)
# Ref: Ebrahim, A., J. A. Lerman, B. O. Palsson and D. R. Hyduke (2013), "COBRApy: COnstraints-Based Reconstruction and Analysis for Python." BMC Syst Biol 7: 74
```

```
import cobra
from cobra import Metabolite, Reaction
from cobra import solvers
solver = solvers.cglpk

# read metabolites list in .mat format consisting of names and KEGG ids of metabolites

mets_db = cobra.io.mat.load_matlab_model("met_database.mat")

#Start: find optimal overall stoichiometry (optStoic)

#create a list of metabolite names

met_names = mets_db.reactions._dict.keys()

#set up the specific problem

#specify bounds for all metabolites

#set the reactants:

#the following set of reactants shows an example where acetyl-CoA is the target product
```

```
known_met = ['C00001','C00080','C01438','C00011','C00237','C00282','C14819','C14818','C00009',
             'C00024','C00014','C00283']

#H2O:C00001    /    Ferric: C14819    /    ACA: C00024    /    SCA: C00091
#H+: C00080    /    Ferrous:C14818    /    OXA: C00036    /    E4P: C00279
#CH4:C01438    /    ATP: C00002       /    G6P: C00092    /    R5P: C00117
#CO2:C00011    /    ADP: C00008       /    PYR: C00022    /    2KG: C00026
#CO: C00237    /    Pi: C00009        /    PEP: C00074    /    F6P: C00085
#H2: C00282    /    H2S: C00283       /    GAP: C00118    /    3PG: C00197
#NH4:C00014    /


#set the bounds for all to greater than zero except known metabolites

for i in met_names:

    if i not in known_met:

        mets_db.reactions.get_by_id(i).lower_bound = -10.0

        mets_db.reactions.get_by_id(i).upper_bound = 10.0


#then set specific bounds for metabolites in the overall stoichiometry

#the following bounds demonstrates a specific case where ferric and CO2 uptakes are fixed to 8
mol and 2.5 mol, respectively
```

```
mets_db.reactions.get_by_id(known_met[0]).lower_bound = -10 #H2O

mets_db.reactions.get_by_id(known_met[0]).upper_bound = 10 #H2O

mets_db.reactions.get_by_id(known_met[1]).lower_bound = -10 #H+

mets_db.reactions.get_by_id(known_met[1]).upper_bound = 10 #H+

mets_db.reactions.get_by_id(known_met[2]).lower_bound = -10 #CH4

mets_db.reactions.get_by_id(known_met[2]).upper_bound = 0 #CH4

mets_db.reactions.get_by_id(known_met[3]).lower_bound = -2.5 #CO2

mets_db.reactions.get_by_id(known_met[3]).upper_bound = -2.5 #CO2

mets_db.reactions.get_by_id(known_met[4]).lower_bound = -10 #CO

mets_db.reactions.get_by_id(known_met[4]).upper_bound = 0 #CO

mets_db.reactions.get_by_id(known_met[5]).lower_bound = 0 #H2

mets_db.reactions.get_by_id(known_met[5]).upper_bound = 0 #H2

mets_db.reactions.get_by_id(known_met[6]).lower_bound = -8 #oxidized electron acceptor

mets_db.reactions.get_by_id(known_met[6]).upper_bound = -8 #oxidized electron acceptor
```

```
mets_db.reactions.get_by_id(known_met[7]).lower_bound = 0 #reduced electron acceptor

mets_db.reactions.get_by_id(known_met[7]).upper_bound = 10 #reduced electron acceptor

mets_db.reactions.get_by_id(known_met[8]).lower_bound = -10 #Pi

mets_db.reactions.get_by_id(known_met[8]).upper_bound = 0 #Pi

mets_db.reactions.get_by_id(known_met[9]).lower_bound = 0.435 #product

mets_db.reactions.get_by_id(known_met[9]).upper_bound = 0.435 #product

mets_db.reactions.get_by_id(known_met[10]).lower_bound = -10 #NH4

mets_db.reactions.get_by_id(known_met[10]).upper_bound = 0 #NH4

mets_db.reactions.get_by_id(known_met[11]).lower_bound = -10 #H2S

mets_db.reactions.get_by_id(known_met[11]).upper_bound = 0 #H2S


#change delta_G constraint from = 0 to <= 0

mets_db.metabolites.get_by_id('dGf')._constraint_sense = "L"

mets_db.metabolites.get_by_id('dGf')._bound = -5
```

```python
#create a dictionary of binary variables (y) corresponding to metabolite i
#in the list met_names and assign 0 or 1 to the binary variable y
#based on the number of C atoms in metabolite i


y_dict={}

for j in met_names:

    try:

        if mets_db.reactions.get_by_id(j).get_coefficient('C') != 0:

            if mets_db.reactions.get_by_id(j).get_coefficient('C') > 10:

                y_dict[j] = 0

            else:

                y_dict[j] = 1

    except:

        y_dict[j] = 0


for j in met_names:
```

```
    try:
        if mets_db.reactions.get_by_id(j).get_coefficient('charge') >= 10000000:
            y_dict[j] = 0
    except:
        pass
for j in met_names:
    try:
        if mets_db.reactions.get_by_id(j).get_coefficient('dGf') >= 10000000:
            y_dict[j] = 0
    except:
        pass
for j in met_names:
    try:
        if mets_db.reactions.get_by_id(j).get_coefficient('R') > 0:
```

```
            y_dict[j] = 0

    except:

        pass

#update the lower and upper bounds of all other metabolites based on the
#values obtained for y

for k in met_names:

    if k not in known_met:

        mets_db.reactions.get_by_id(k).lower_bound = y_dict[k] *
mets_db.reactions.get_by_id(k).lower_bound

        mets_db.reactions.get_by_id(k).upper_bound = y_dict[k] *
mets_db.reactions.get_by_id(k).upper_bound


#convert the non-linear problem to linear problem

#create a dictionary of corresponding metabolites in model

s_dict = {}
```

```
#create a dictionary of binary variables corresponding to each metabolite
yb_dict = {}

#initiate a new constraint to represent sum(yi) <= n
cons_b = Metabolite('y_const')
cons_b._constraint_sense = "L"
cons_b._bound = 12

#initiate the conversion of non-linear (s_i * y_i) to linear (w_i = s_i * y_i) problem
for i in met_names:
    #initiate the binary variable y_i
    yb_dict[i] = 'y_'+i
    y_i = Reaction(yb_dict[i])

    #declare y_i to be an integer
```

```
y_i.variable_kind = "integer"

#set the flux bounds for y_i
if i in known_met:
    y_i.lower_bound = 1.0
    y_i.upper_bound = 1.0
elif y_dict[i] == 0:
    y_i.lower_bound = 0.0
    y_i.upper_bound = 0.0
else:
    y_i.lower_bound = 0.0
    y_i.upper_bound = 1.0

#initiate the w_i variable
s_dict[i] = 's_'+i
```

```
s_i = Reaction(s_dict[i])

#set the flux bounds for w_i

s_i.lower_bound = mets_db.reactions.get_by_id(i).lower_bound

s_i.upper_bound = mets_db.reactions.get_by_id(i).upper_bound

#add four additional constraints for each metabolite

cons1 = Metabolite('c1_'+i) # w_i + M * y_i >= 0

cons2 = Metabolite('c2_'+i) # -w_i + M * y_i >= 0

cons3 = Metabolite('c3_'+i) # w_i - M * y_i - s_i >= -M

cons4 = Metabolite('c4_'+i) # -w_i - M * y_i + s_i >= -M

#set the non-equality of each constraint

cons1._constraint_sense = "G"

cons2._constraint_sense = "G"

cons3._constraint_sense = "G"
```

```
cons4._constraint_sense = "G"

#set the bounds of constraints

cons1._bound = 0

cons2._bound = 0

cons3._bound = -10

cons4._bound = -10

#obtain the stoichiometry of metabolite i in the database

w_i = mets_db.reactions.get_by_id(i)

#associate stoichiometries to each constraint

w_i.add_metabolites({cons1:1,cons2:-1,cons3:1,cons4:-1})

s_i.add_metabolites({cons3:-1,cons4:1})

y_i.add_metabolites({cons1:10,cons2:10,cons3:-10,cons4:-10,cons_b:1})
```

```
#add new rxns to model
mets_db.add_reaction(s_i)

#add the new binary variable to model
mets_db.add_reaction(y_i)

#objective coeffs
w_i.objective_coefficient = 0.0
y_i.objective_coefficient = 0.0
s_i.objective_coefficient = 0.0

if i not in known_met:
    w_i.objective_coefficient = 1.0
else:
    if i == 'C00237':
```

```
            w_i.objective_coefficient = -1.0
        elif i == 'C01438':
            w_i.objective_coefficient = -1.0
        elif i == 'C00011':
            w_i.objective_coefficient = 0.0
        elif i == 'C00118':
            w_i.objective_coefficient = 0.0
        else:
            w_i.objective_coefficient = 0.0

#solve optStoic
fi = open("data.txt", "w")
epsilon = 1e-9
n = 0
new_solution = []
```

```
old_solution = ['y_C00001']

while new_solution != old_solution:
    FBA_soln = mets_db.optimize()
    n = n + 1
    old_solution = new_solution
    new_solution = []
    lp = solver.create_problem(mets_db, objective_sense="minimize")
    solver.set_parameter(lp, "tolerance_feasibility", 1e-9)
    print('')
    print('Iteration'+str(n))
    fi = open("data.txt", "a")
    fi.write( 'Iteration'+str(n) + "\n"  )

    for i in range(len(FBA_soln.x_dict)):
```

```
        if FBA_soln.x_dict.keys()[i] in yb_dict.values() and FBA_soln.x_dict.values()[i] == 1:

            new_solution.append(FBA_soln.x_dict.keys()[i])

        if FBA_soln.x_dict.keys()[i] in met_names and (FBA_soln.x_dict.values()[i] > epsilon or 
FBA_soln.x_dict.values()[i] < -epsilon):

            try:

                met_dg = mets_db.reactions.get_by_id(FBA_soln.x_dict.keys()
[i]).get_coefficient('dGf')

            except:

                met_dg = 1000


            met_id = FBA_soln.x_dict.keys()[i]

            met_stoic = abs(FBA_soln.x_dict.values()[i])

            dg_stoic = met_stoic * met_dg

            print met_id,FBA_soln.x_dict.values()[i],dg_stoic

            fi.write( str(met_id) + " ")
```

```
        fi.write( str(FBA_soln.x_dict.values()[i]) + " ")
        fi.write( str(dg_stoic) + "\n"  )
    fi.close()

    #add a constraint
    cons_ic = Metabolite('int_cut_'+str(n))
    cons_ic._constraint_sense = "L"
    cons_ic._bound = cons_b._bound - 1
    for i in new_solution:
        y_i = mets_db.reactions.get_by_id(i)
        y_i.add_metabolites({cons_ic:1})

#end
```
